# POST-COVID-19 ASSESSMENT: HEALTH LITERACY AND PREVENTION PRACTICES IN LTC FACILITIES IN INDIA: PROVIDER PERSPECTIVE

**DOI:** 10.1093/geroni/igac059.1782

**Published:** 2022-12-20

**Authors:** Shantha Balaswamy, Raja Samuel, Rufus Singh

**Affiliations:** Ohio State University, Dublin, Ohio, United States; Madras School of Social Work, Chennai, Tamil Nadu, India; Madras School of Social Work, Chennai, Tamil Nadu, India

## Abstract

There were no governmental issued guidelines directed specifically towards Long-Term Care residential facilities (LTCF) for older adults in India during COVID-19 pandemic (Rajagopalan 2020) and no data has been collected on care for resident in NGO’s and private facilities (WHO, 2021). Further, studies of level of knowledge of providers on health indicators of residents, health literacy and risks factors of contracting the virus is absent. In India, older adults who live in LTCF have higher rates of chronic diseases (hypertension, diabetes, respiratory, heart problems), live in crowded communal space, less access to medical care for underlying health conditions, and have untrained, multiple caregivers, thereby putting them at increased risk for exposure and contracting the COVID-19 virus, and significant risk of infection, illness and mortality. LTCF includes NGO’s, government funded old age homes and private paid retirement homes and is considered a multi-residence housing facility. This exploratory study, using online survey of providers of approximately 125 LTC facilities, examines the providers general health literacy level, prevention practices at their facility interventions, demands and rewards of caring for residents during COVID. Preliminary results less than 20% of providers tracked prevalence of illness, health symptoms, infection rate, hospitalization, mortality rate, or post-recovery. Majority reported difficulty in accessing vaccination, getting residents vaccinated, and accessing resources ( food/ medicine/sanitizers) and problem coordinating care due to staffing shortage. Only a small percent use and track health and psychological assessments of residents. Findings have implications for advocating for standardization care and support National and State LTCF policy.

